# Private Weakly-Random Sequences from Human Heart Rate for Quantum Amplification

**DOI:** 10.3390/e23091182

**Published:** 2021-09-08

**Authors:** Maciej Stankiewicz, Karol Horodecki, Omer Sakarya, Danuta Makowiec

**Affiliations:** 1 Institute of Mathematics, Faculty of Mathematics, Physics and Informatics, University of Gdańsk, Wita Stwosza 57, 80-308 Gdańsk, Poland; maciej@stankiewicz.edu.pl; 2 International Centre for Theory of Quantum Technologies (ICTQT), University of Gdańsk, 80-308 Gdańsk, Poland; 3 Institute of Informatics, National Quantum Information Centre, Faculty of Mathematics, Physics and Informatics, University of Gdańsk, Wita Stwosza 57, 80-308 Gdańsk, Poland; osakarya@sigma.ug.edu.pl; 4 Institute of Theoretical Physics and Astrophysics, Faculty of Mathematics, Physics and Informatics, University of Gdańsk, Wita Stwosza 57, 80-308 Gdańsk, Poland; danuta.makowiec@ug.edu.pl

**Keywords:** quantum randomness amplification, weak randomness test, heart rate

## Abstract

We investigate whether the heart rate can be treated as a semi-random source with the aim of amplification by quantum devices. We use a semi-random source model called ε-Santha–Vazirani source, which can be amplified via quantum protocols to obtain a fully private random sequence. We analyze time intervals between consecutive heartbeats obtained from Holter electrocardiogram (ECG) recordings of people of different sex and age. We propose several transformations of the original time series into binary sequences. We have performed different statistical randomness tests and estimated quality parameters. We find that the heart can be treated as a good enough, and private by its nature, source of randomness that every human possesses. As such, in principle, it can be used as input to quantum device-independent randomness amplification protocols. The properly interpreted ε parameter can potentially serve as a new characteristic of the human heart from the perspective of medicine.

## 1. Introduction

Randomness is an essential resource in many applications of everyday life [1]. As prominent examples, there comes a generation of passwords and tokens for online banking: most transactions are secured by random numbers sent to the user’s telephone for authentication purposes. Both public and symmetric key encryption are based on a significant amount of randomness. Quantum key distribution developed in the last decades [2] is also based on the access to a true random number generator. The choice of measurements applied to devices needs to be unknown to the eavesdropper, i.e., random with respect to him/her. For these reasons, the privacy of randomness is often the target of hackers’ attacks and can be considered one of the “Achilles’ heels” of security systems. Designing hardware that produces private randomness is a risky task, as recent attacks on such devices show [3].

As a cure for the lack of both reliable and uniform sources of randomness, there comes the theory of the so-called extractors [4,5]. One can apply a deterministic function for two or more weakly random sources, which extracts (outputs) nearly uniform and private randomness. There are two major models of the weak source of randomness: the Santha–Vazirani (SV) [6] source and the $H_{\min}$ source [7]. Note the following two facts: First, the weak sources (at least two of them) have to be mutually statistically independent so that the extractors work. Second, due to the seminal result of Miklos Santha and Umesh Vazirani, it is known that from a single such source that amplification is impossible [6]. In a recent breakthrough, Roger Colbeck and Renato Renner have shown that a single weak source of randomness can be amplified by the use of quantum devices [8,9]. This seminal result has been improved—just two devices are needed for this task both in the case of the quantum [10,11,12,13] and the so-called no-signaling adversary [14,15,16,17].

A significant problem in the use of pairs of devices is the vulnerability to attack, which is based on correlating a weak source with device [18]. One solution that proposes a partial way out is called “privatization” of a weak source of randomness. One chooses a weak source as an emitter of data that is hard to control for anyone, including eavesdroppers. Even though the signal comes publicly to the honest parties, due to its prior unpredictability to the eavesdropper, its randomness can be amplified by quantum protocols [19]. This solution, however, has a drawback. Between the weak source and quantum amplifier, there is a long chain of detecting devices, each of which can be in principle replaced by a prefabricated source of randomness correlated to the quantum one.

In this manuscript, we propose a novel direction looking for both hard-to-predict and hard-to-replace sources of randomness. Our aim is thus amplification of such a source via a quantum device. An example that we focus on in this manuscript is the heart rate. Let us here note that in this case, quantum amplification is a natural option to consider. Indeed, the heart appears to be a unique private source of weak randomness of a human. This is because it seems plausible that a fully uncorrelated source of weak randomness from the same person’s body, decoupled from his/her heartbeat, may not exist. Furthermore, using two persons’ hearts to extract private randomness by classical extractors may not be a good idea, as the same characteristics of the human heart as a muscle can potentially lead to unwanted correlations. Therefore, considering the quantum application may lead to more straightforward implementation in the future than the classical one, based on two uncorrelated weak sources.

The heart rate has been considered for quite a long time as a potential source of randomness in several applications [20,21,22,23]. However, it is known that fluctuations of heart rate display scale-invariant non-Gaussian probability density functions [24] what maps in the observed so-called 1/f scaling [25] and multifractality [26] of heart rate. The most pronounced long-range correlations are commonly linked to interactions with the respiratory system (3 to 9 beats), vascular system (9 to 24 beats), and other intrinsic regulatory systems (more than 24 beats) [27]. Therefore, it has been discussed whether the heart rate is of the stochastic origin or if it is governed by some deterministic nonlinear system [28]. However, to our knowledge, there is neither proof of its true randomness nor a firm disproof. Both would be interesting practically. The proof would imply a reliable source of weak randomness. Simultaneously, disproof would potentially lead to fast (compressed) heart rate data transfer. This feature would be important in case of an emergency when a patient’s data are being sent to the hospital. Although this type of source is treated as random in several applications, it has been checked recently that raw data from heart rate repositories do not pass tests for randomness [29]. However, as we aim to amplify the randomness of the heart rate, we do not need it to be uniformly random from the beginning.

We perform tests to check to what extent heart rate can be treated as SV-source. One cannot directly perform the test of randomness due to the nature of the definition of SV-source. We perform partial tests. We also provide software for testing data for the SV conditions, which may be of independent interest.

Notably, there are classical extractors [5,30] which are applicable in the quantum regime. These hash functions output randomness secure against a quantum adversary [31] when fed with two independent sources of weak randomness. Given that it is hard to build a device correlated to someone’s heart rate, these extractors are applicable in our context.

The solution we propose is ecological as opposed to, e.g., random number generators based on radioactive decay [32]. It is also cheap and easily accessible due to the rapid development of wearable electronic devices. One could consider other bioinspired sources of randomness, but the heart-based seems to be the easiest to detect [33]. Therefore, we push further the limit of the work in [34], where it was noted that private randomness is always present in the message. Here, we report that the one that is present in the message sender itself can be amplified. Note that we base our experiments on the randomness on which the sender’s consciousness has a small impact. Indeed, humans are known to create non-random sources when they, e.g., type [35].

While a low data rate can be seen as a drawback, there always is an option of randomness expansion [13]. Amplification results in a small amount of high-quality randomness called a seed. The expansion method uses the seed to generate an arbitrarily long random sequence.

As an interesting side effect, our research can also lead to new findings that are important from the medical point of view. Indeed, we present certain cryptographic parameters for healthy volunteers. Comparing these parameters with those obtained from persons with particular diseases can potentially lead to a novel characterization of the latter. As a method of preprocessing the heartbeat, we propose looking for the specific periodic behavior in the data. In this approach, we “split” the signal into two sequences: a periodic one and a more random one. The periodicity can be stronger or weaker. In the case of patients with certain diseases, its strength may potentially better describe particular illnesses.

## 2. Materials and Methods

We focus on a model of semi-random sources called Santha–Vazirani source [6]. An ε-Santha–Vazirani (ε-SV) source is characterized as follows. Let $S=(S_{1},S_{2},\ldots )$ be a source described by sequence of binary random variables $S_{i}$. By $s=(s_{1},s_{2},\ldots )$ we will denote arbitrary long bit string produced by the source *S* (realization of the source and therefore random variables). We say that the source is an ε-SV if for all *i*

(1) $$\frac{1}{2}-\varepsilon \leq P\left(S_{i+1}|S_{i},\ldots ,S_{1},E\right)\leq \frac{1}{2}+\varepsilon$$

where *E* represents an arbitrary random variable prior to $S_{1}$ that can influence the source. It is easy to see that for ε=0 the bits are fully random and for ε=1/2 they can even be deterministic.

The most straightforward example of the SV source is the source of tossing a fake coin with distribution {1/2+ε,1/2−ε}. Moreover, the ε-SV source has been characterized in [9] as a mixture of specific permutations of this distribution. As such, it does not pass standard randomness tests, such as Dieharder [36]. However, as we argue, it appears to be random enough to be amplified to uniformly random bit-string if combined with another independent device, e.g., one can use the amplification given in [19] (although we do not need privatization of an SV source, as the SV source of our choice is practically private).

### 2.1. Used Data and Their Preprocessing

The ECG recording displays the electrical activity of the heart muscle as a sequence of events: the contraction of the atria followed by the contraction of the ventricles. Therefore, the shape of the ECG curve is systematically analyzed by cardiologists in order to determine heart performance. If the ECG displays correct properties, then the heartbeat is classified as normal. When any deviation is observed, the beat is classified further according to the specifics of the observed deviation. The most pronounced peak in the normal ECG curve is denoted by R, and it corresponds to the time of the beginning of the contraction of ventricles. The time distance between two consecutive ventricle contractions, which are normal-to-normal heart contractions, is referred to as RR interval. The RR interval is used as a measure of the length of the first heartbeat.

Twenty-four-hour Holter ECG recordings during a normal sleep–wake cycle were obtained from healthy volunteers without any known cardiac history. The Holter recordings were analyzed using Del Mar Reynolds Impresario software and screened for premature, supraventricular, and ventricular beats, missed beats, and pauses. These data were annotated correspondingly by the automatic system. The entries which were not annotated as normal (N) were neglected.

While such preprocessing have little physiological significance, it is satisfactory for the cryptographic goal. We will refer to these data as preprocessed for cryptographic purposes.

The data preprocessed in such a way were organized as follows. Each person has a separate text file with sex, age, and start time of measurement encoded into the file name. In each file, after the header, there are four columns: number of the observation, time of observation, length of RR interval, and annotation. As Del Mar software used to obtain data has a 128 Hz sampling frequency (which means approximately 8 ms resolution), the RR interval column entries are represented as a limited set of rational numbers.

The signals had to be preprocessed by an experienced cardiologist to obtain a physiological meaning of the data. It occurs that the heart’s activity during the day is affected by some external factors due to brain stimulation via interactions with the environment, which makes the signal analysis extremely difficult. For this reason, only the nocturnal part of the Holter record has been thoroughly corrected manually by cardiologists and annotated correspondingly.

The hours of sleep were identified for each signal individually in order to detect the day–night transition properly. A six-hour period, covering the longest RR-intervals, was extracted as the nocturnal period. Perturbations in signals—artifacts or not normal-to-normal RR-intervals—were edited as follows. Perturbations consisting of less than five consecutive RR-intervals were replaced by the median estimated from the last seven normal RR-intervals; other perturbations were deleted. Eventually, the nocturnal signals were constructed from at least 20,000 RR intervals.

We will denote preprocessed sequence of RR intervals as ${\left\{d_{i}\right\}}_{i=1}^{n}$. We will refer to this data as to manually preprocessed.

#### 2.1.1. Discretization

As we need a binary sequence for purpose or randomness amplification (and testing SV source parameter), we have to apply some form of discretization. We have tested three classes of parameterized function that maps a string of rational numbers ${\left\{d_{i}\right\}}_{i=1}^{n}$ to a binary sequence ${\left\{s_{i}\right\}}_{i=1}^{n}$.

The first one assigns deceleration of the heart rate to 0 and acceleration to 1 in the following way:

(2) $$s_{i}=\left\{\begin{array}{cc} 0: & d_{i}\geq d_{i-1}+\eta_{1}\\ 1: & \mathrm{otherwise} \end{array}\right.,$$

where $\eta_{1}$ is an offset parameter. The second one outputs zero if the change in heart rate is rapid (above threshold $\eta_{2}$) and one if it is slow using the equation

(3) $$s_{i}=\left\{\begin{array}{cc} 0: & |d_{i}-d_{i-1}|\geq \eta_{2}\\ 1: & \mathrm{otherwise} \end{array}\right..$$

The last one, in some sense, take into consideration the monotonicity of three consecutive heartbeats in the following way:

(4) $$s_{i}=\left\{\begin{array}{cc} 0: & d_{i}\geq d_{i-1}\geq d_{i-2}\vee d_{i}\leq d_{i-1}\leq d_{i-2}\\ 1: & \mathrm{otherwise} \end{array}\right..$$

During the initial phase of our experiment, we discovered that the best results are obtained using the first method of discretization with $\eta_{1}=0$. Our choice of discretization method was based on ϵ estimation for the ϵ-SV source model. We will provide a detailed description of how the estimation works in Section 2.2. The choice of $\eta_{1}$ was straightforward. We observed that for all $\eta_{1}\neq 0$, the proportion of zeros and ones in the discretized sequence was much further away from 1 than in $\eta_{1}=0$ case. Therefore, the results presented in the remaining part of the paper will use the first method of discretization with $\eta_{1}=0$.

#### 2.1.2. Cutting Out Trends

First, let us note that the data from the heart have partially periodic behavior by the nature of the source: after several consecutive accelerations, there needs to come, sooner or later, several slowdowns. This implies that this source cannot satisfy the SV condition: a too-long sequence of ones denoting accelerations cannot appear in the data. On the other hand, in *n* long bit-string ε-SV source it has probability ${\left(\frac{1}{2}-\varepsilon \right)}^{n}>0$. However, we can modify the source by cutting out the acceleration and deceleration parts. This is known as cutting out trends. This approach is parametrized by pair of natural numbers (i,j). It means that we cut out *i* consecutive signals of acceleration and the first next *j* consecutive signals of slow down, after which we look for the next *i* accelerations and *j* decelerations and so on through the whole sequence. In our experiment, we consider only the case i=j∈{3,…,6}. Let us clarify that it is also possible to use values i≠j, and our software implementation allows such cases. Nonetheless, we decide to concentrate on the cases where i=j since we think there is some form of symmetry in heart rate (for example, in acceleration and deceleration).

As we will see, this method resulting in lowering the rate of the source yields a more random one. By doing so, we base on the fluctuations of the heart rate. Note that this approach is different from other considered in literature [37] that are based on fluctuations of the measurement devices such as a camera. Such methods based on fluctuations or noise of the signal recorded by the measurement devices can be prone to attacks that use malicious measurement devices. On the other hand, our approach uses the main part of the signal (heart rate) and presumably can be resistant to such attack vectors. It can be done by using several different measurement devices from different manufacturers and comparing the results.

### 2.2. Randomness Testing Method Details

In this section, we will describe the idea behind our testing method called SVTest implemented in the software we use.

Our goal is to estimate ε from Equation (1). We will do it by estimating values $\varepsilon_{h}$ where $h\in \{0,\ldots ,h_{\max}\}$ are history lengths (condition lengths). We will postpone reasoning how to choose $h_{\max}$ to the later part of the paper. Using the definition of conditional probability and more verbose notation we can rewrite Equation (1) to obtain value $\varepsilon_{h}$ in explicit way

(5) $$\varepsilon_{h}:=\max_{s_{n-h+1},\ldots ,s_{n+1}\in \left\{0,1\right\}}\;\left|\frac{P(s_{n+1},s_{n},s_{n-1},\ldots ,s_{n-h+1})}{P(s_{n},s_{n-1},\ldots ,s_{n-h+1})}-\frac{1}{2}\right|.$$

We should point out here that the above definition makes sense only for random number generating devices (that are modeled by probability distributions). As we treat the device as a black box, we have only access to some binary sequence $s=(s_{1},s_{2},\ldots ,s_{n})$ that is outputted by the device (that can be seen as a realization of the device’s probability distribution). Because of this, our approach to approximate $\varepsilon_{h}$ is to use frequencies to estimate probabilities from Equation (5). By doing this, we obtain

(6) $${\tilde{\varepsilon }}_{h}:=\max_{w_{h}}\left|\frac{\frac{{\left|s\right|}_{w_{h}}}{n-h}}{\frac{{\left|s\right|}_{w_{h}^{\prime }}}{n-h+1}}-\frac{1}{2}\right|\underset{n\to \infty }{\approx }\max_{w_{h}}\left|\frac{{\left|s\right|}_{w_{h}}}{{\left|s\right|}_{w_{h}^{\prime }}}-\frac{1}{2}\right|$$

where $w_{h}:=\left(x_{1},x_{2},\ldots ,x_{h}\right)$ is binary sequence, $w_{h}^{\prime }:=\left(x_{1},x_{2},\ldots ,x_{h-1}\right)$, and ${\left|a\right|}_{b}$ denotes number of occurrence of substring *b* in string *a*. Here, by ${\tilde{\epsilon }}_{h}$ we denote the experiment-driven value to make it distinct from the theoretical one $\epsilon_{h}$.

#### Similar Tests Used in Previous Research

The so-called serial test from the seminal NIST test suite [38] uses a similar approach to our method. The serial test is focused on checking the frequency of all overlapping *h*-bit sequences. The three main differences are that

- frequencies are calculated instead of conditional values,
- average square deviation is used instead of maximal absolute deviation, and
- cyclic approach is used at the end of the sequence.

Furthermore, the NIST test suite was developed to check pseudo-random sequences to use them directly in classical algorithms. This approach demands the sequence to be almost perfectly random. On the other hand, as we apply the quantum randomness amplification method, it is enough that the sequence is partially random, assuming that we know the threshold ε.

Another, quite a similar testing method was presented by Martinez et. al. [39] that is based on the Borel-normality criterion [40], but in their approach, overlapping is not used. For us, it is interesting that they claim that the longest history length used should be of the order of $\log_{2}\left(\log_{2}\left(n\right)\right)$.

### 2.3. Identifying ε from a Realization of a Source of Weak Randomness

We propose a method to estimate ε of the underlying SV source given finite realization of this source. Our method can be applied to any other source of weak randomness, and thus may be of interest itself. It is, however, important to stress that our approach is heuristic and can be further developed.

First, we note that given a finite sequence of data, we cannot expect ε-SV conditions to be satisfied for all lengths of histories. A trivial observation is that the maximal history length *h* in a sequence of length *n* satisfies

(7) h≤log(n)−1

(see Lemma 1 below).

**Lemma** **1.** *For the sequence of data $s_{n}$ of length n, the maximal length of history h that satisfies ε-SV Condition (1) with ε<1/2 satisfies*

(8) h≤log(n)−1

**Proof.**  We note that the Condition (1) implies that $2^{h+1}\leq n-\left(h+1\right)$. This is because it implies that all sequences of length h+1 (of history and one bit history of which is considered) should appear in the sequence $x_{n}$. The term h+1 on r.h.s. appears because the last position at which such sequence can start is n−h−1, as it has length h+1 and should be a subsequence of $x_{n}$. Taking logarithm on both sides we obtain h+1≤log(n−h−1)≤logn, thus the assertion follows. □

One can ask if there exists a realization which saturates the above inequality. We give the affirmative answer by observing that the so-called De Bruijn sequences have this property [41]. For any $n=2^{k}$, the De Bruijn sequence $d_{k}$ of length *n* satisfies ideally, i.e., with ε=0 the ε-SV conditions given in Equation (1) up to history of length logn−1=k−1. Unlike in our considerations, in De Bruijn’s construction one assumes cyclic boundary conditions, i.e., that $d_{k}\left[n\right]$ is neighbor of $d_{k}\left[1\right]$. We give the first two exemplary sequences below.

(9) $$d_{2}=0011$$

(10) $$d_{3}=00010111$$

For example, in the sequence $d_{2}$, “0” appears the same number of times as “1”. The same holds for sequences of pairs of bits, each appearing once (sequence “10” is obtained from the cyclic condition).

However, for some sequences, one can observe non-zero ε for histories of shorter length than log(n)−1. However, one needs to attribute single ε to the source. It is rather plausible that the longest histories correspond to events with no statistical meaning as they appear only a few times (or even once) in the complete sequence. We, therefore, propose to use the weighted average, defining ε for the sequence $s_{n}$ as

(11) $$\tilde{\varepsilon }\left(s_{n}\right):=\frac{1}{w(\left\lfloor \log_{2}\left(n\right)\right\rfloor -1)}\sum_{i=0}^{\lfloor \log_{2}\left(n\right)\rfloor -1}\frac{{\tilde{\varepsilon }}_{i}\left(s_{n}\right)}{\left(i+1\right)}$$

with $w\left(h\right)=\sum_{i=0}^{h}\frac{1}{i+1}$.

### 2.4. Software Description

We now describe in Algorithm 1 an algorithm for attributing epsilons for a given history length ($\varepsilon_{h}$) that can be computed from a bit-string. Note that the bit-string need not be a representation of the heart rate, and hence the algorithm can be used in other contexts.
**Algorithm 1:** Estimation of epsilons.
 **Data**: Annotated data file from Holter device, maximal history length parameter $h_{max}$ **Result**: Sequence of $\varepsilon_{i}$ for $i\in \{0,\ldots ,h_{max}\}$ _**1**_  Read appropriate RR intervals from data file; _**2**_  Generate binary sequence from RR intervals using chosen discretization; _**3**_  Optionally: perform cutting out trends subroutine; _**4**_  Count the number of occurrences of each binary substring of length up to $h_{max}+1$; _**5**_  Calculate estimated epsilons given in Equation (6);

In the first step, we read into memory sequence of RR intervals ${\left\{d_{i}\right\}}_{i=1}^{n}$. Furthermore, we only take into account the ones that are valid according to annotations (see the beginning of Section 2.1 for details).

In the second step, the sequence of rational numbers ${\left\{d_{i}\right\}}_{i=1}^{n}$ is changed into binary string ${\left\{s_{i}\right\}}_{i=1}^{n}$ according to chosen discretization. Exemplary classes of functions that can be used to discretize are discussed in Section 2.1.1.

In the third step, the so-called cutting out trends is performed. It is not mandatory as it reduces the number of bits, but in some cases, it can be beneficial due to smaller epsilons in the shorter resulting sequence. We discuss this method in Section 2.1.2.

In the fourth step, we count the number of occurrences of each binary string of length not larger than $h_{max}+1$ as a consecutive substring of our binary data string. It is done in such a way that substrings can overlap. We do not assume cyclicity, i.e., the end of the data string is not considered as following the beginning of it. For example, in the sequence 001011011, substring “0” appears four times, substring “1” five times, substring “00” one time, substring “01” three times, substring “10” two times, substring “11” two times, substring “000” zero times, and so on.

In the fifth step, we use appropriate numbers of occurrences from the previous step to calculate epsilons according to the formulas from Section 2.2. At this point the program outputs sequences ${\left\{\varepsilon_{i}\right\}}_{i=1}^{h_{max}}$.

An additional step is to obtain single epsilon from the above sequence of epsilons. As it was easier to analyze epsilons’ behavior, we perform that step separately, outside of our program. Our suggested way of calculating single, final epsilon is described in Section 2.3.

#### Implementation

Our software is implemented in the C programming language. We used several optimization features to speed up the analysis. As we are interested in overlapping substrings, we treated sub-sequences as natural numbers and used bit shift operation to process the next bit. We also count the number of occurrences of each substring only once and use it to calculate $\varepsilon_{i}$ for all *i* at the very end instead of processing each history length separately. Furthermore, as a number of occurrences of the sequences starting with “1” can be deduced from appropriate sequences that start with “0” and previously calculated shorter ones, we can omit it, reducing running time and amount of used memory.

Such optimization was sufficient because of (the characteristic for our research) type and a moderate amount of data. On the other hand, some other features can be implemented in future software versions if needed. For example, reading it whole at once for extremely large data sets can be problematic because of the limited amount of memory. In such a case, reading files in smaller portions can be a better solution. Furthermore, a multi-threaded version of the program could be considered. Finally, we can even think of reading data from the stream and estimating epsilons on the fly, for example, in real-time applications.

## 3. Results

We will present experiments taking two points of view: cryptographic and medical. For medical analysis, we will consider data that are preprocessed by medical experts (see Section 2.1). For cryptographic purposes, we consider raw data with less physiological meaning. The latter approach is justified, as, in a potential application, there will be no place for an expert to preprocess the raw data before their randomness is quantumly amplified. Indeed, it would be not only impractical but also against the approach of device-independent processing, which assumes no trust to third parties.

### 3.1. Identifying ε of Exemplary Raw Data for Cryptographic Purpose

Considering heartbeat as a source, we lack a large bulk of data. To overcome this, we consider a merged file of data from 118 persons as if they come from a much longer period for a single person. The heart rate data, which we consider here, come from an 8-h long recording of many persons (data merged together taken from 66 women and 52 men). The main reason to process a large amount of data is to find the best value of parameters (i,j) for the “cutting out trends” method in subsequent experiments. In Table 1, we show the values of the ε based on the merged file.

Namely, we show behavior of two values of ε obtained for six patterns of cutting out trends (i,j)∈{(2,2),…,(6,6)} (the first value (i,j)=(0,0) denotes the case without cutting out trends). The values of the pattern are shown in Table 1, in its first row. As we can see, cutting out trends significantly lowers the amount of data (from *n* to m≤n). Indeed, as depicted in the second row and second column, the initial data contain m=n= 24,447,658 bits, while the subsequent values are much lower up to m= 120,997 for the (6,6) pattern. In the third row, there are values of ε computed on the sub-sequences of the merged file. The sub-sequences are the same length as the output of the cutting out trends for the corresponding pattern. That is, e.g., in the result of cutting out trends of pattern (2,2), the data became as short as m= 3,820,864 bits, so the ε is computed from the first *m* bits of the original merged file. The fourth row shows the values of the $\varepsilon_{cut}$, i.e., the value of ε computed on the merged file processed by the cutting out trends procedure with a corresponding pattern. The bottom row shows the difference between the values of ε and $\varepsilon_{cut}$. Although cutting out larger patterns (i,i) yields better ε, we focus on the case (3,3) because the output string is too short for i>3. Note that cutting out trends with pattern (2,2) increases the value of ε, and thus should not be taken into account.

**Figure 1:**
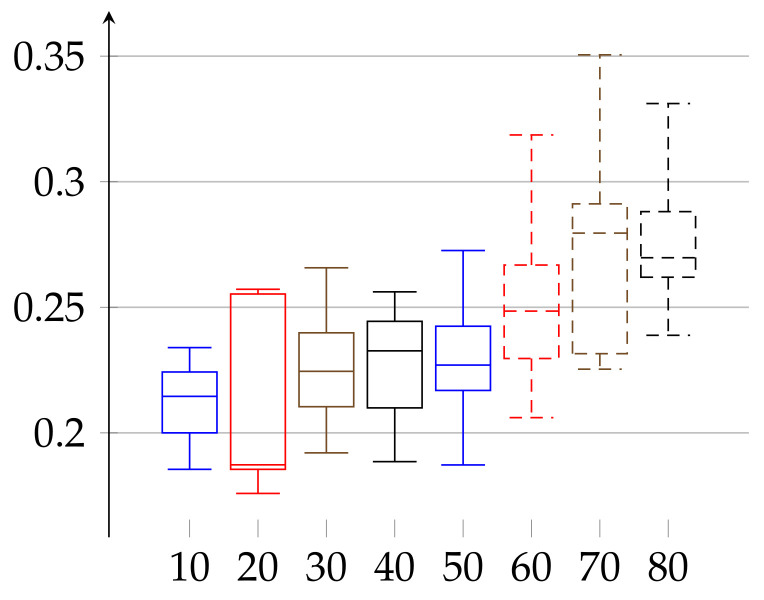
Values of *ε* for the group of women, aged 19–89, shown in increasing order of age (without cutting trends). The bottom line of the rectangular shape denotes the first quartile, the top one denotes the third quartile, and the middle one denotes the second quartile (median). The minimal and maximal values (the 0th and 4th quartiles) are depicted as top and bottom whiskers for each age group.

**Figure 2:**
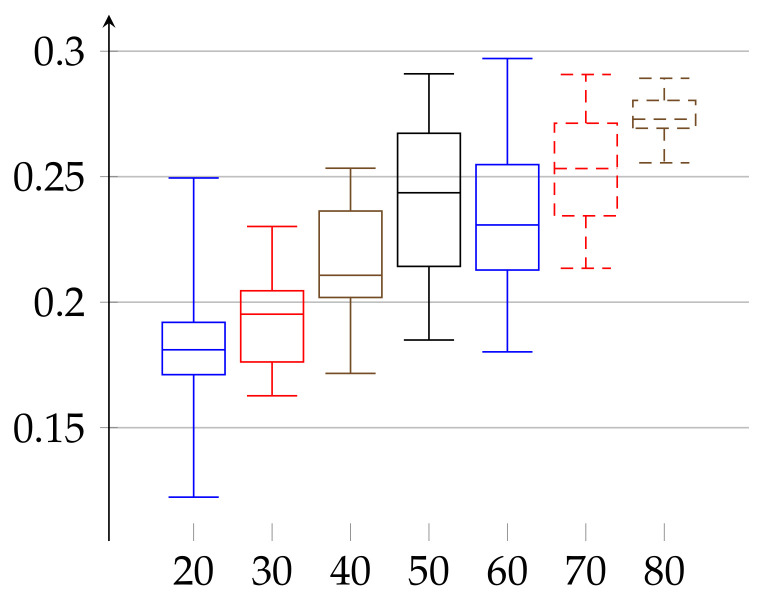
Values of *ε* for the group of men, aged 21–88, shown in increasing order of age. The bottom line of the rectangular shape denotes the first quartile, the top one denotes the third quartile, and the middle one denotes the second quartile (median). The minimal and maximal values (the 0th and 4th quartiles) are depicted as top and bottom whiskers for each age group.

We are ready to describe the experimental results of epsilon for two groups of volunteers. The first group consists of 66 women aged 19–89 years. The second consists of 52 men aged 21–88 years. We divided each group into subgroups of persons with age belonging to interval [10∗j,10∗(j+1)) for j∈{1,…,8} for women and [10∗k,10∗(k+1)) with k∈{2,…,8} for men. The age groups consisted of 10: 3, 20: 9, 30: 11, 40: 7, 50: 6, 60: 9, 70: 9, and 80: 12 elements in case of women and 20: 11, 30: 10, 40: 8, 50: 3, 60: 5, 70: 9, and 80: 6 in case of men. In Figure 1 and Figure 2, we describe the computed ϵ according to definition given in Equation (11). In case of women the value of $\epsilon^{\left(w\right)}\in \left[0.17588,0.35052\right]$ and the median has growing tendency. The data of women with age below 20 may have low statistical meaning due to the low number of persons in this group. These trends should be confirmed with more persons in a given age ranges. Similarly, in the case of men, the median of epsilon also has an increasing tendency with age and is in the range $\epsilon^{\left(m\right)}\in \left[0.12232,0.29707\right]$ (note that the values for men with age in the range 50–59 have low statistical meaning, due to the low number of volunteers in this age).

**Figure 3:**
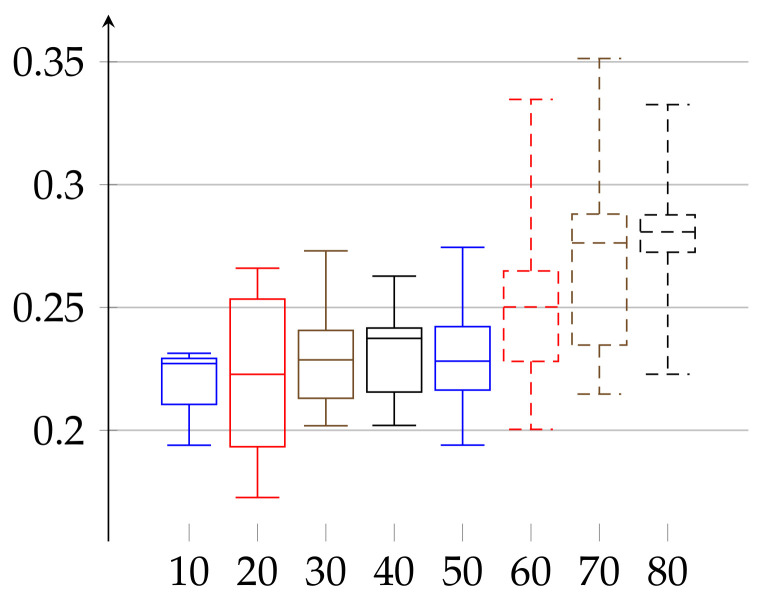
Values of *ε* for the group of women aged 21–88 shown in increasing order of age for the same length of data (cut to the minimal length among all persons). Depiction of statistical quantities (0–4th quartile) as described in caption of Figure 1.

**Figure 4:**
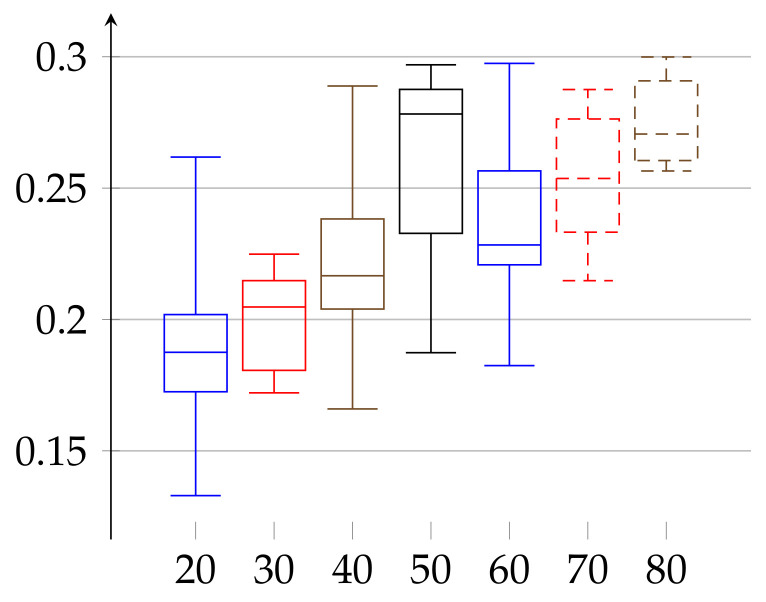
Values of *ε* for the group of men aged 21–88 shown in increasing order of age for the same length of data (cut to the minimal length among all persons). Depiction of statistical quantities (0–4th quartile) as described in caption of Figure 1.

In the second experiment, depicted in Figure 3 and Figure 4, we show the results for the ϵ achieved for the same initial amount of data for each person. In the previous experiment (Figure 1 and Figure 2), because we work on real heart rate from volunteers, obtained by Holter device and preprocessed by Del Mar software, the amount of data (number of recorded valid RR intervals) can be very different for each person. Therefore, the resulting binary sequences have different lengths for different individuals. One could argue that this difference in length (in some cases even twice as much) could be a reason for unnecessary bias in our epsilon estimation. Therefore, we have decided to perform this second experiment. In it, we truncated the data (by removing an appropriate number of bits from the end) for each person so that the resulting binary sequences have the same length for each person. In this case, $\epsilon^{\left(w\right)}\in \left[0.17262,0.35135\right]$ and $\epsilon^{\left(m\right)}\in \left[0.13295,0.29990\right]$. In that case, the value of median of ϵ has growing tendency for both women and men.

**Figure 5:**
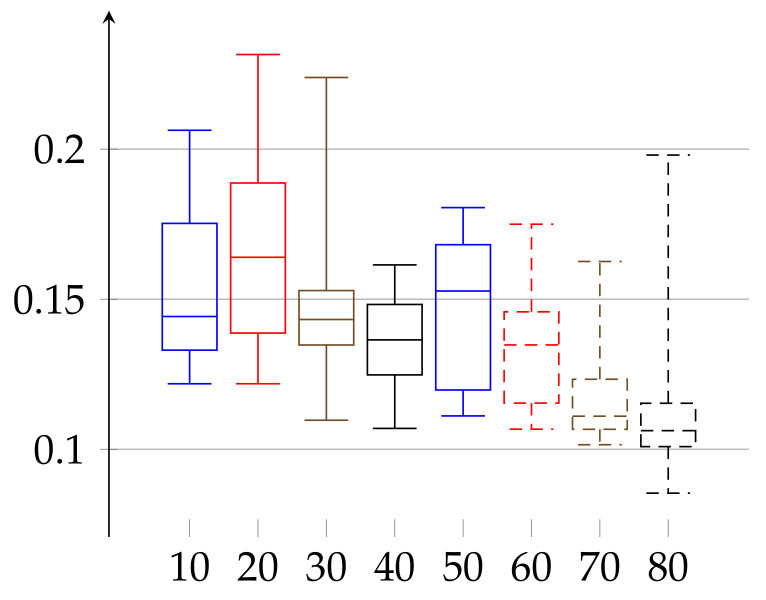
Values of *ε* for the group of women aged 19–89 shown in increasing order of age, after post-processing according to cutting out trends with pattern (3, 3). Depiction of statistical quantities (0–4th quartile) as described in caption of Figure 1.

**Figure 6:**
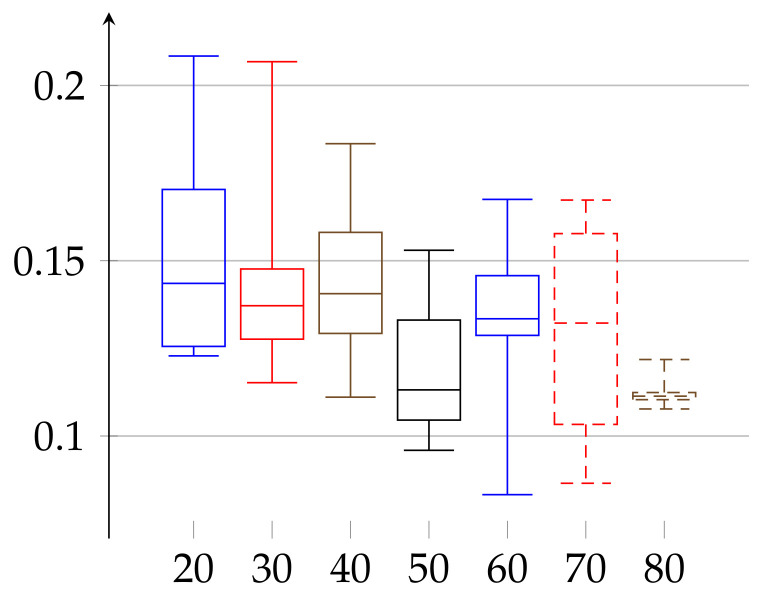
Values of *ε* for the group of men aged 21–88 shown in increasing order of age, after post-processing according to cutting out trends with pattern (3, 3). Depiction of statistical quantities (0–4th quartile) as described in caption of Figure 1.

In the final experiment (see Figure 5 and Figure 6, respectively) we apply the postprocessing of cutting trends described in the above. As a result, ϵ lowers in both cases to reach the ranges $\epsilon^{\left(w\right)}\in \left[0.08539,0.23153\right]$ for women and $\epsilon^{\left(m\right)}\in \left[0.08327,0.20838\right]$ for men.

### 3.2. Identifying ε of Exemplary Manually Pre-Processed Data for Medical Purpose

Although we concentrate on the cryptographical aspect of the heart rate data, we extend our findings to the ones with strictly medical meaning. This is because the ϵ-SV can, in principle, be treated as a parameter with possible novel meaning from the medical point of view. To confirm its relevance, one should compare its value with those obtained from persons with particular diseases. Here, we show the values of $\tilde{\epsilon }$ for healthy persons.

**Figure 7:**
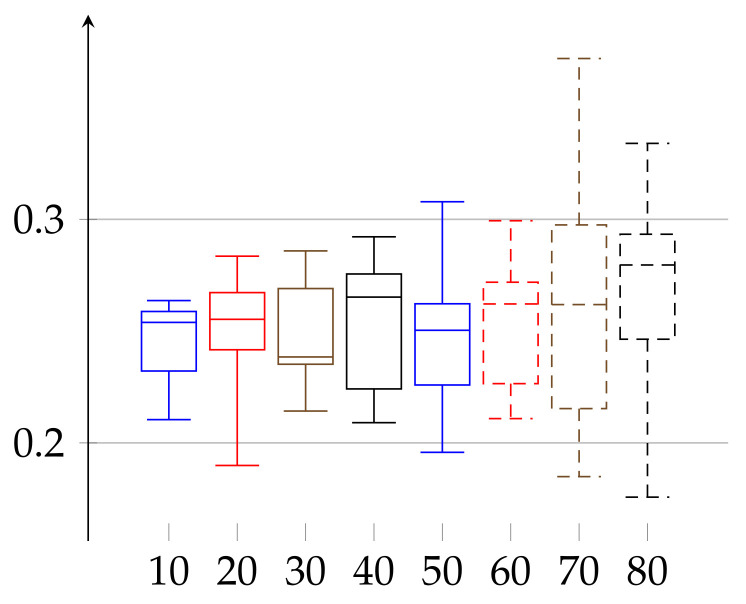
Values of *ε* for the group of women aged 19–89 shown in increasing order of age, after manual preprocessing by a medical expert. Depiction of statistical quantities (0–4th quartile) as described in caption of Figure 1.

**Figure 8:**
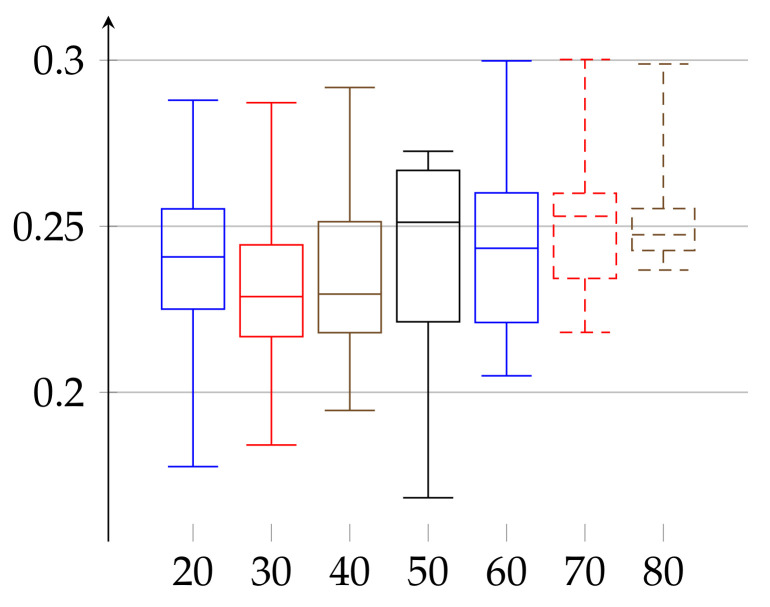
Values of *ε* for the group of men aged 21–88 shown in increasing order of age, after manual preprocessing by a medical expert. Depiction of statistical quantities (0–4th quartile) as described in caption of Figure 1.

In this medical approach, we have studied manually preprocessed data of 190 persons. The first group consists of 88 women aged 19–89 years, and the second consists of 102 men aged 21–88 years. We divided each group into subgroups of persons with age belonging to interval [10∗j,10∗(j+1)] for j∈{1,…,8} for women and [10∗k,10∗(k+1)] with k∈{2,…,8} for men. The age groups consisted of 3 women of age 10–19, which we denote as 10: 3 and accordingly, 20: 15, 30: 11, 40: 13, 50: 13, 60: 12, 70: 10, and 80: 11 elements in case of women and 20: 17, 30: 12, 40: 20, 50: 19, 60: 15, 70: 12, and 80: 7 in case of men. On Figure 7 and Figure 8 we describe the computed ϵ according to definition given in Equation (11) for each sex, respectively. In the case of women, the value of epsilon reads $\epsilon^{\left(w\right)}\in \left[0.17573,0.33397\right]$ and in case of men the epsilon is in the range $\epsilon^{\left(m\right)}\in \left[0.16825,0.30020\right]$, which resembles the results obtained from cryptographic signals, but there is no observed age dependency on the age group medians.

### 3.3. Comparison of Different Experimental Methods

**Figure 9:**
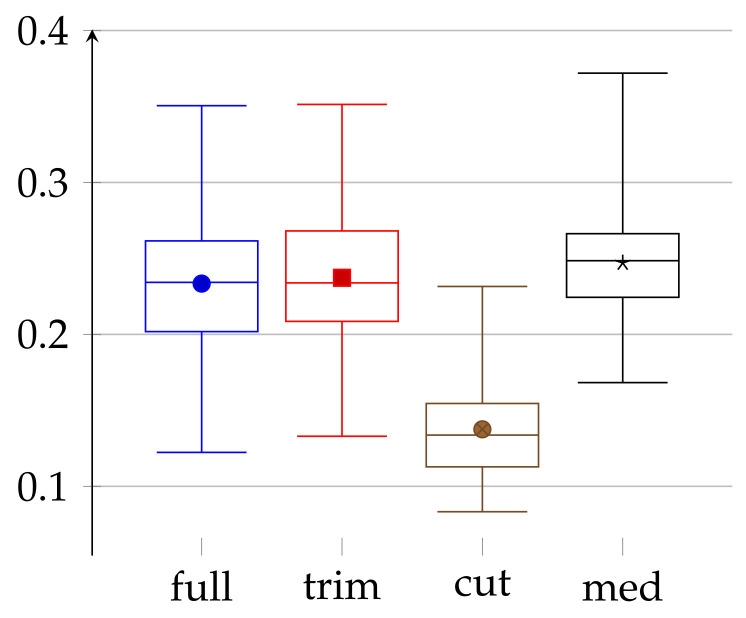
Collective values of *ε* for all persons (regardless of age and sex) for four experiments presented in our work. For description see Section 3.3 Depiction of statistical quantities (0–4th quartile) as described in caption of Figure 1. Additionally, bold dots in the figure represent average values.

To conclude the main part of our experiment, we present in Figure 9 a comparison of different methods that we have used during our studies. In this figure, we analyze four different approaches: full—referring to the full data, trim—full data trimmed to the same length, cut—full data after “cutting out trends”, and med—manually preprocessed data. The first three are discussed in Section 3.1; the fourth one is described in Section 3.2.

In this comparison, we can make a few interesting observations. First, trimming data to the same length has a marginal impact on the values of epsilons. This may be because this trimming removes only a small portion of data. Second, epsilons for medical data do not differ much from the one obtained in the “full” approach. On the one hand, in the medical case, the amount of data (for each person) is, in fact, a few times smaller, but on the other hand, the data are carefully chosen (both by taking into consideration only sleep time and manually removing incorrect values). Finally, the “cut” approach, which uses “cutting out trends”, gives significantly better results. It, therefore, shows that the carefully chosen preprocessing can impact the quality of obtained randomness, however, at a price of a lower amount of data. Our “cutting out trends” pre-processing is successful because the heartbeat has natural periodicity built-in, which is obviously predictable, i.e., not random with respect to the adversary.

## 4. Discussion

In this manuscript, we have considered the heart rate as a weak source of randomness in the context of quantum methods of its amplification. As one of these methods is an amplification of the ε-Santha–Vazirani source, we have checked if the heart rate can be modeled as such a source. We have proposed the way to attribute ε to finite data according to which longer histories have a lower weight than shorter ones. As this proposition is heuristic, other ways could be considered. We have also proposed preprocessing of the signal, called cutting trends, which is natural in the context of heart rate data. It decreased the value of epsilon by half. It would be interesting to see if other preprocessing or discretization of the signal can lead to lower values of ε.

In the experimental part of this manuscript, we have considered detailed data and estimated its ε. Interestingly, its value does not strongly depend neither on the age nor sex of volunteers, and after cutting trends, it is of the order of ≈0.13. It seems then to be a parameter of a heart as a human’s muscle. One can also consider a heart-based Santha–Vazirani parameter ε≠1/2 as a precondition of life of every heart possessing being, including humans.

Fitting a quantum randomness amplification scheme to the above result would be the next step towards a device which is not vulnerable to attack based on the correlation between source and device [18,19].

From the medical point of view, the values of ε obtained from the data with physiological meaning do not differ much from the raw data-derived one. Moreover, the observed lack of dependence of ϵ on age suggests that there could be a universal pattern of a healthy heart rhythm. Therefore, it would be interesting in the future to compare the value of ε obtained from healthy persons, which we have shown, with the one from persons with particular diseases. This would give additional medical meaning to a cryptographic parameter leading to an interesting interplay between the two apparently distant domains. Further, studying the pattern of the cut out trend (in our case, from the cryptographic point of view, the most successful was (3, 3)) can be of further interest also from the medical point of view.

## Figures and Tables

**Table 1:** Values of epsilons.

Pattern	(0, 0)	(2, 2)	(3, 3)	(4, 4)	(5, 5)	(6, 6)
**size (m)**	24,447,658	3,820,864	1,191,328	488,968	241,072	120,997
***ε***	0.21884	0.20976	0.20972	0.190349	0.201891	0.185005
***ε_cut_***	NA	0.22117	0.134322	0.105582	0.104898	0.0934031
***ε* − *ε_cut_***	NA	−0.01141	0.0753987	0.0847669	0.0969931	0.0916016

## Data Availability

The spreadsheet containing all results obtained from our program and further calculations is available in Supplementary Materials of this work (for details, see Appendix A). Source heart rate data files can be sent upon request. Our program is closed source software.

## Supplementary Materials

The following are available at https://www.mdpi.com/article/10.3390/e23091182/s1, Sheet 1: all persons without cutting, Sheet 2: all persons without cutting, Sheet 3: all persons using 3,3 cuting, Sheet 4: all persons manually prepro, Sheet 5: merged, Sheet 6: single person, Sheet 7: comparison.

## Appendix A. Detailed Numerical Results

In this section, we will describe the form of detailed results of the numerical experiments. For the sake of the reader, we provide the results in the form of a spreadsheet attached to this paper as Supplementary Materials. The spreadsheet file, named “results.ods”, is divided into several sheets describing different approaches that we used.

In the first of the four sheets in the file, we present results for all persons separately in different settings. At the top, each column represented one person what is indicated by the corresponding name of the input data file. In these sheets, for the convenience of the reader, we used the following color-coding. In yellow, there are input file names, input file sizes, and numbers of data entries obtained from the input files after preprocessing (number of bits used in randomness test). Below that, in cyan, there are all epsilons estimated by our SVTest program, where each row corresponds to a different length of history used in the test. Then, in green, there are single values of epsilons for each person calculated according to Equation (11). At the bottom, in red, we presented various statistics for sets of persons grouped by sex and age intervals. The statistics are described in the main text and depicted in the appropriate figures. Additionally, texts in bold font are headers describing different fields. Finally, all other values with a white background are local variables used in calculations and can be omitted (unless the reader wants to understand the details of the statistics calculations fully).

In Sheet 1, we used full data files for each person using only necessary preprocessing without cutting out trends. Statistics for this approach are presented in Figure 1 and Figure 2.

In Sheet 2, we used the same data as above, but we trimmed the number of entries to be the same for each person. This allows us to analyze data without taking into consideration potential epsilons bias that can be a result of different numbers of bits. Statistics for this approach are presented in Figure 3 and Figure 4.

In Sheet 3, we applied cutting out trends procedure (using parameters (3, 3)) to the full data from Sheet 1. Statistics for this approach are presented in Figure 5 and Figure 6.

In Sheet 4, we present results for the medical approach that involves manual preprocessing. Appropriate statistics are presented in Figure 7 and Figure 8.

In Sheet 5, we present results of various approaches (cutting out trends with different parameters and original file trimmed to analogical lengths) obtained from the large input files consisting of data from all persons concatenated together. This approach allows us to simulate the behavior of the heart rate in a very long (possibly multi-day) measurement. Part of these results is summarized in Table 1.

In Sheet 6, we show results of different approaches (similar to Sheet 5 for a single person.

Finally, In Sheet 7, we show combined results of all persons (from all age groups and both sex) for different approaches. Then numbers 1 to 4 corresponds to Sheets 1–4 from above. Statistics for this approach are presented in Figure 9.
